# Current News

**Published:** 1896-01

**Authors:** 


### Current News.

### MEETING IN WESTERN PENNSYLVANIA.

A MEETING of the local societies of Western Pennsylvania will be held January 21, 22, and 23.

*Executive Committee.*—J. G. Templeton, H. W. Arthur, O. L. Hertig, Geo. Shidle, C. M. George, and J. A. Libbey, chairman.

